# Post-lingual deafness: benefits of cochlear implants vs. conventional hearing aids

**DOI:** 10.1590/S1808-86942012000200019

**Published:** 2015-10-20

**Authors:** Aline Gomes Bittencourt, Liliane Satomi Ikari, Ana Adelina Giantomassi Della Torre, Ricardo Ferreira Bento, Robinson Koji Tsuji, Rubens Vuono de Brito Neto

**Affiliations:** a MD, ENT (Fellow in ear and skull base surgery at Hospital das Clínicas in the Medical School of the University of São Paulo – FMUSP, Brazil); b MD (Specialized in ENT) Medical School of the University of São Paulo – FMUSP, Brazil; c Professor (Professor of ENT Practice at the Medical School of the University of São Paulo – FMUSP, Brazil); d PhD in ENT at the University of São Paulo (Assisting Physician in the ENT Practice Course at Hospital das Clínicas in the Medical School of the University of São Paulo – FMUSP, Brazil); e Professor (Associate Professor in the ENT Practce Course at the Medical School of the University of São Paulo – FMUSP, Brazil). Disciplina de Clínica Otorrinolaringológica do Hospital das Clínicas da Faculdade de Medicina da Universidade de São Paulo – FMUSP, Brasil

**Keywords:** cochlear implants, deafness, hearing aids, hearing loss, sensorineural

## Abstract

The technological advances in cochlear implants and processing strategies have enabled subjects affected by severe to profound hearing loss to hear sounds and recognize speech in various different degrees. The variability of hearing outcomes in subjects with post-lingual deafness has been significant and cochlear implant indications have been extended to include an ever larger population.

**Objective:**

This paper aims to look into the groups of post-lingual deafness patients to find where cochlear implants have yielded better outcomes than conventional hearing aids.

**Materials and Methods:**

Review the literature available on databases SciELO, Cochrane, MEDLINE, and LILACS-BIREME. The publications selected for review were rated as A or B on evidence strength on the day of the review. Their authors analyzed and compared hearing aids and cochlear implants in populations of post-lingually deaf patients. Study Design: Systematic review.

**Results:**

Eleven out of the 2,169 papers searched were found to be pertinent to the topic and were rated B for evidence strength. Six studies were prospective cohort trials, four were cross-sectional studies and one was a clinical trial.

**Conclusion:**

The assessment done on the benefits yielded by post-lingually deaf subjects from cochlear implants showed that they are effective and provide for better results than conventional hearing aids.

## INTRODUCTION

The technological advances in cochlear implants and processing strategies have enabled subjects affected by severe to profound hearing loss to hear sounds and recognize speech in various different degrees^1^. The variability of hearing outcomes in subjects with post-lingual deafness has been significant, and the most relevant factors to predict good outcome are short period of pre-implant hearing deprivation and some residual hearing^2^. That is why cochlear implant indications have been extended to include an ever larger population^1^.

Several studies have been published within the last few years comparing the hearing outcomes obtained by severe and profound hearing loss patients using conventional hearing aids or cochlear implants^3^. This paper aims to look into the groups of post-lingual deafness patients to find where cochlear implants have yielded better outcomes than conventional hearing aids, having papers published in the literature as reference.

## MATERIALS AND METHODS

This is a systematic review carried out upon the medical literature after a search conducted on June of 2010, including papers written in Portuguese, English and Spanish. The search for relevant references was done on databases SciELO, Cochrane, MEDLINE, and LILACS–BIREME. The publications selected for review were rated as A or B on evidence strength on the day of the review. Their authors analyzed and compared hearing aids and cochlear implants in populations of post-lingually deaf patients. The following search terms (keywords and delimiters) were used: *cochlear implants/cochlear implantation; hearing aid; therapy; prognosis; comparative study* and the corresponding translated terms in various combinations.

Some of the included studies might be biased, as they were written by cochlear implant manufacturers^4^, ^5^, ^6^.

## RESULTS

Eleven out of the 2,169 papers searched were found to be pertinent to the topic and were rated B for evidence strength (four were rated 2b^1^, ^7^, ^8^, ^9^ and seven 2c^2^, ^3^, ^4^, ^5^, ^6^, ^10^, ^11^). No A-rated publications were found. Six of the selected studies were prospective cohort trials, four were cross-sectional studies and one was a clinical trial (Table 1).

**Table 1:** Characteristics of the studies included in this review.

Study	Year of Publication	Study Type	Sample Size	Age Range	Assessment Tool	Evidence Rating
Spillmann & Dillier	1990	Cross-sectional	63	14 to 50 years	Mininal Auditory Capabilities (MAC)	2c
Snik et al.	1997	Prospective cohort	18	4 to 10 years	Pure-tone and speech audiometry	2c
Hamzavi et al.	2001	Prospective cohort	37	23 to 76 years	Hochmaier, Schultz and Moser sentence test (HSM)	2b
MED EL	2001	Prospective cohort	63	18 & older	Hearing in Noise Test (HINT) and City University New York (CUNY) Sentence Test	2c
UK Cochear Implantation		Prospective cohort	84	40 to 58 years	Bamford-Kowal-Bench (BKB) Sentence Test e City University New York (CUNY) Sentence Test	2b
Ching et al.	2004	Cross-sectional	21	25 to 81 years	Bamford-Kowal-Bench (BKB) Sentence Test	2b
UK Cochear Implantation	2004	Prospective cohort	316	16 to 82 years	Audiometry, Speech intelligibility test, Mark III Health Utilities Index	2c
Mo et al.	2004	Cross-sectional	134	19 to 85 years	Performance Inventory for Profound and Severe loss	2c
Mo et al.	2004	Cross-sectional	179	19 to 85 years	Patient Quality of Life Form (PQLF) Index Relative QuestionnaireForm(IRQF), Short Form 36 (SF-36) Hopkins Sympton Check List (HSCL-25) Performance Inventory for Profound and Severe Loss (PIPSL)	2c
Looi et al.	2008	Prospective cohort	30	36 to 80 years	Music test battery	2c
Poissant et al.	2010	Clinical trial	25	37 to 92 years	Speech perception testing	2b

Spillmann & Dillier^9^ applied a test battery called *Minimal Auditory Capabilities* (MAC) to groups of conventional hearing aid users and users of single-channel and multichannel cochlear implants. Based on outcome comparison, the authors intended to predict which patients should not be deemed good candidates for cochlear implants. The MAC test, albeit not a standardized procedure, was considered as a useful tool in advising and selecting patients for cochlear implant use.

Snik et al.^10^, using speech perception tests, assessed seven children users of cochlear implants and another eleven who used conventional hearing aids to conclude that the scores on the presented phonemes were similar for both groups.

In 2001, a study by MED-EL Ltda.^4^ (Innsbruck, Austria) looked at speech perception and compared pre-implantation scores of conventional hearing aid users to their scores six months after they started using cochlear implants. The authors carried out two series of subgroup analyses: pre and post-lingually deaf; and (2) time of hearing loss in post-lingually deaf patients (mean 25 years of age). The mean difference (pre/post) for post-lingually deaf patients was 62% in silent environments, and patients with less than 25 years of hearing loss yielded greater benefits from cochlear implants than those who had experienced hearing loss for over 25 years (71% *vs*. 53% respectively). In noisy environments, post-lingually deaf patients with 25 years or less of hearing loss also performed better.

Ching et al.^1^ observed 21 adult patients wearing cochlear implants Nucleus 22 (Cochlear Ltd., Lane Cove, Australia) or Nucleus 24 (Cochlear Ltd.). Twelve patients made combined use of conventional hearing aids and cochlear implants, while nine did not use conventional hearing aids after implantation. The combined use of cochlear implant and hearing aid was compared to cochlear implant or conventional hearing aids alone. Patients wearing both devices performed significantly better on speech tests, on functional performance questionnaires, and made many fewer mistakes as they had to locate a sound source when compared to patients wearing either cochlear implants or conventional hearing aids. The authors used the *Bamford-Kowal-Bench (BKB) Sentence Test* in noisy situations to measure speech perception and showed a significant benefit for cochlear implant users (CI user mean score was 39, while conventional hearing aid users' was 2, *p* < 0.001).

Mo et al.^2^ evaluated 134 patients (75 CI users and 59 hearing aid users) aged between 19 and 85 years of age using the *Performance Inventory for Profound and Severe Loss*. The CI group scored better (*p* < 0.01) on *Visual Cues* (USV)*, Intensity* (INT), *Response to Auditory Failure* (RAF). No significant difference was found on *Environmental Sounds* (ES), *Visual Cues* (USNV) and *Personal* (PER). Note that CI patients had mean preoperative hearing in their best ears of 113 dB. On the other hand, conventional hearing aid users had mean hearing of 82 dB. Nevertheless, at the end of the study the cochlear implant group had better social hearing capabilities.

Mo et al.^3^ compared 84 adult users of cochlear implants to three groups of severe/profound hearing loss patients: 19 patients accepted for implantation, but on whom surgery was not performed (subgroup non-CI-A); 16 candidates to CI whose hearing loss was not severe enough for them to use cochlear implants (subgroup non-CI-B); and 60 conventional hearing aid users. Five questionnaires were applied: *Patient Quality of Life Form (PQLF)*, *Index Relative Questionnaire Form* (*IRQF)*, *Short Form 36 (SF-36)*, *Hopkins Symptom Check List (HSCL-25)* and *Performance Inventory for Profound and Severe Loss (PIPSL)*. The most significant differences were observed on quality-of-life and degree of depression and anxiety (HSCL-25) between the groups of cochlear implant patients and subgroup non-CI-A. Cochlear implant patients had significantly less depression and anxiety than their non-implanted counterparts. The only significant difference between cochlear implant and conventional hearing aid users was elicited on questionnaire SF-36, in which the CI group scored better.

Hamzavi et al.^7^ measured speech perception in severe/profound hearing loss patients before implantation and 12 months into follow-up in cochlear implant and conventional hearing aid users. The authors also looked into alterations between 12 and 36 months after implantation on silent and noisy conditions using the Hochmair, Schultz and Moser (HSM) sentence test. They found that cochlear implant patients had a mean improvement of 90% on their pre/post-implantation test scores, while conventional hearing aid users improved by 37%. Monosyllabic word test scores improved by 43% for CI users and by 19% for conventional hearing aid users. In two years, the HSM scores in silent conditions improved by 16% for CI users and by 0% for conventional hearing aid users. In noisy conditions, conventional hearing aid users failed to show improvement, whereas CI users improved on all noise levels.

In 2004, the *United Kingdom Cochlear Implant Study Group*^5^ (UKCISG) studied 316 patients with severe/profound hearing loss who wore either conventional hearing aids or cochlear implants. The authors assessed the cost for the United Kingdom National Health Service to provide and maintain cochlear implants, as estimated by the *Mark III Health Utilities Index*. The study showed acceptable cost-effectiveness for cochlear implantation in all assessed patients. It further indicated median cost-effectiveness for cochlear implants in patients affected by hearing loss for over 30 years and who were then using conventional hearing aids. Additionally, age was a factor that worsened CI cost-effectiveness, once it is estimated that older individuals have fewer years of life ahead of them.

On that same UKCISG^6^ cohort, 84 patients were tested for speech perception using the *Bamford-Kowal-Bench (BKB) Sentence Test and the City University New York (CUNY) Sentence Test* before implantation and nine months since in patients with profound hearing loss. Results have shown improved scores on both measurements nine months after surgery [BKB: *Marginal hearing aid users* (MHU) = 44.0 (95% IC 37–51); AVGN: MHU = 31.0 (95% IC 26–37)].

According to Poissant et al.^8^, there is no significant difference in the ability to understand speech in silent or noisy conditions between younger and older cochlear implant users (*p*<0.05). The author observed fewer cases of depression among older cochlear implant users and of loneliness in both older and younger subjects. Older cochlear implant users were not more depressed or lonely than their counterparts with mild to moderate hearing loss who used conventional hearing aids.

Assuming that conventional hearing aid users would have better musical perception skills than cochlear implant individuals, Looi et al.^11^ looked into the performance of subjects with similar levels of hearing loss who used either of the devices. The results for both groups were nearly identical for rhythm tests, with the conventional hearing aid group marginally outperforming the cochlear implant group in pitch and melody tests. Nonetheless, there was no difference between the groups in their ability to identify musical instruments or ensembles.

## DISCUSSION

Hearing loss is a problem of significant prevalence in the population that affects one's personality and socialization, possibly leading to isolation and reclusion. Hearing test results of cochlear implant patients vary. Some can communicate without the aid of orofacial reading and even talk on the phone, while others can only hear sounds from the environment, alarm buzzers or beepers, and improve their lip reading skills.

Several changes are taking place as to how cochlear implant candidates are chosen, often driven the increasingly global and dynamic pace of knowledge acquisition. Patients with low frequency residual hearing in the implanted ear can undergo less traumatic procedures, avoid endocochlear trauma, and use shorter or perimodiolar electrode bundles, thus allowing them to preserve their hearing at lower frequencies and even use conventional hearing aids and cochlear implants in a combined fashion on one ear.

Uma questão importante é a comprovação científica de que o IC pode trazer benefícios superiores aos das próteses auditivas convencionais, que justifiquem o procedimento cirúrgico, em termos de riscos operatórios e encargos psicossociais e financeiros ao paciente, sua família e ao sistema de saúde. Thus, cochlear implant candidates must be chosen as a function of observed outcomes and their predictive factors.

According to the Brazilian Association of Otorhinolaryngology, adolescents aged 12 and over and adults with post-lingual deafness are candidates for unilateral or bilateral cochlear implants if they meet the following criteria: severe or profound bilateral sensorineural hearing loss; open form sentence recognition of 50% or less; hearing aids on both ears and patient adequately motivated to use cochlear implants and undergo speech and hearing rehabilitation.

After a broad systematic review, we have been able to find methodologically distinct studies, mainly when outcomes are analyzed. However, they make it clear that cochlear implants are superior when compared to conventional hearing aids in several aspects. Some studies, such as the UKCISG^5^ cohort and the paper by Hamzavi et al.^7^, compared patients in an ideal form while they were using hearing aids and later as they receive cochlear implants, using the same tests pre and postoperatively.

It has been found that cochlear implants allow for improvements in speech understanding and on quality-of-life, aside from significantly easing one's insertion into the social and professional worlds and reducing the onset of depression and feelings of loneliness^1^, ^3^, ^4^, ^6^, ^7^, ^8^, ^10^. Shorter hearing deprivation time has been singled out as a factor connected to better outcomes^4^, ^6^.

## CONCLUSION

The analysis of the studies included in this systematic review has enabled us to conclude that cochlear implants allow for better performance in post-lingually deaf patients when compared to conventional hearing aids as verified by audiological tests, improved quality-of-life, and overall procedure cost-effectiveness.

## References

[1] Ching TY, Incerti P, Hill M (2004). Binaural benefits for adults who use hearing aids and cochlear implants in opposite ears. Ear Hear..

[2] Mo B, Harris S, Lindbaek M (2004). Cochlear implants and health status: a comparison with other hearing-impaired patients. Ann Otol Rhinol Laryngol..

[3] Mo B, Lindbaek M, Harris S, Rasmussen K (2004). Social hearing measured with the Performance Inventory for Profound and Severe Loss: a comparison between adult multichannel cochlear implant patients and users of acoustical hearing aids. Int J Audiol..

[4] MED-EL. Summary of safety and effectiveness data. P000025. FDA; 2001. URL: http://www.fda.gov/cdrh/pdf/P000025b.pdf. Accessed February 2007

[5] UK Cochlear Implant Study Group (2004). Criteria of candidacy for unilateral cochlear implantation in postlingually deafened adults II: cost-effectiveness analysis. Ear Hear..

[6] UK Cochlear Implant Study Group (2004). Criteria of candidacy for unilateral cochlear implantation in postlingually deafened adults I: theory and measures of effectiveness. Ear Hear..

[7] Hamzavi J, Franz P, Baumgartner WD, Gstöettner W (2001). Hearing performance in noise of cochlear implant patients versus severely-profoundly hearing-impaired patients with hearing aids. Audiology..

[8] Poissant SF, Beaudoin F, Huang J, Brodsky J, Lee DJ (2008). Impact of cochlear implantation on speech understanding, depression, and loneliness in the elderly. J Otolaryngol Head Neck Surg..

[9] Spillmann T, Dillier N (1990). Comparison of hearing aids and cochlear implants in profoundly and totally deaf persons. Br J Audiol..

[10] Snik AF, Vermeulen AM, Brokx JP, van den Broek P (1997). Long-term speech perception in children with cochlear implants compared with children with conventional hearing aids. Am J Otol..

[11] Looi V, McDermott H, McKay C, Hickson L (2008). Music perception of cochlear implant users compared with that of hearing aid users. Ear Hear..

